# Carotid Artery Stenting and Blood–Brain Barrier Permeability in Subjects with Chronic Carotid Artery Stenosis

**DOI:** 10.3390/ijms18051008

**Published:** 2017-05-08

**Authors:** Arkadiusz Szarmach, Grzegorz Halena, Mariusz Kaszubowski, Maciej Piskunowicz, Michal Studniarek, Piotr Lass, Edyta Szurowska, Pawel J. Winklewski

**Affiliations:** 1 2nd Department of Radiology, Medical University of Gdansk, Gdansk 80-210, Poland; a.szarmach@gumed.edu.pl (A.S.); eszurowska@gumed.edu.pl (E.S.); 2 Department of Cardiovascular Surgery, Medical University of Gdansk, Gdansk 80-210, Poland; g.halena@gumed.edu.pl; 3 Department of Economic Sciences, Faculty of Management and Economics, Gdansk University of Technology, Gdansk 80-210, Poland; fraglesior@wp.pl; 4 1st Department of Radiology, Medical University of Gdansk, Gdansk 80-210, Poland; m.piskunowicz@gumed.edu.pl (M.P.); m.studniarek@gumed.edu.pl (M.S.); 5 Department of Diagnostic Imaging, Medical University of Warsaw, Warsaw 03-242, Poland; 6 Department of Nuclear Medicine, Medical University of Gdansk, Gdansk 80-210, Poland; plass@gumed.edu.pl; 7 Institute of Human Physiology, Medical University of Gdansk, Gdansk 80-210, Poland; 8 Department of Clinical Sciences, Institute of Health Sciences, Pomeranian University of Slupsk, Slupsk 76-200, Poland

**Keywords:** computed tomography perfusion, carotid artery stenosis, blood–brain barrier, permeability surface, cerebral blood flow, cerebral blood volume, mean transit time, time to peak

## Abstract

Failure of the blood-brain barrier (BBB) is a critical event in the development and progression of diseases such as acute ischemic stroke, chronic ischemia or small vessels disease that affect the central nervous system. It is not known whether BBB breakdown in subjects with chronic carotid artery stenosis can be restrained with postoperative recovery of cerebral perfusion. The aim of the study was to assess the short-term effect of internal carotid artery stenting on basic perfusion parameters and permeability surface area-product (PS) in such a population. Forty subjects (23 males) with stenosis of >70% within a single internal carotid artery and neurological symptoms who underwent a carotid artery stenting procedure were investigated. Differences in the following computed tomography perfusion (CTP) parameters were compared before and after surgery: global cerebral blood flow (CBF), cerebral blood volume (CBV), mean transit time (MTT), time to peak (TTP) and PS. PS acquired by CTP is used to measure the permeability of the BBB to contrast material. In all baseline cases, the CBF and CBV values were low, while MTT and TTP were high on both the ipsi- and contralateral sides compared to reference values. PS was approximately twice the normal value. CBF was higher (+6.14%), while MTT was lower (−9.34%) on the contralateral than on the ipsilateral side. All perfusion parameters improved after stenting on both the ipsilateral (CBF +22.66%; CBV +18.98%; MTT −16.09%, TTP −7.62%) and contralateral (CBF +22.27%, CBV +19.72%, MTT −14.65%, TTP −7.46%) sides. PS decreased by almost half: ipsilateral −48.11%, contralateral −45.19%. The decline in BBB permeability was symmetrical on the ipsi- and contralateral sides to the stenosis. Augmented BBB permeability can be controlled by surgical intervention in humans.

## 1. Introduction

Spontaneously hypertensive/stroke-prone rats with unilateral carotid artery occlusion and a high-salt diet develop white-matter damage similar to that seen in vascular cognitive dementia in humans [1]. Development of white-matter damage in these animals is a multiphase process. Initially, chronic hypertension and the related cerebral vessel lumen narrowing result in loss of normal autoregulation, hypoperfusion, and hypoxia. Subsequently, hypoxia augments the production of hypoxia inducible factor-1α (HIF-1α), leading to oxidative stress and an inflammatory state. This is followed by production of matrix metalloproteinases that disrupt the tight junctions and extracellular matrix resulting in the breakdown of the blood–brain barrier (BBB). Eventually, vasogenic oedema accelerates tissue hypoxia, myelin damage and oligodendrocyte death [1].

In humans, functionally impaired but viable tissue may exist in areas of chronic cerebral ischaemia with a combination of misery perfusion and reduced cerebral metabolism [2,3]. The benefits of the carotid artery corrective procedures (e.g., endarterectomy, endovascular stenting or transluminal angioplasty) are well known for prevention from ischaemic brain stroke [4,5,6]. Preoperatively impaired cognitive function occasionally improves after carotid artery surgery. The reversible cognitive impairment is related to a state of reduction in metabolism and downregulation of cortical neurotransmitter receptors in response to a severe reduction in brain perfusion [7,8]. BBB abnormalities have been described in patients with small-vessel disease secondary to hypertension and diabetes [9]. Increased BBB permeability was also reported in patients with broad white matter hyperintensities and symptoms suggestive of subcortical ischaemic vascular disease [10]. Ischaemic insult can exacerbate vascular abnormalities, especially subcortical BBB permeability in the presence of cerebral small vessel disease [11]. It is, however, not yet known whether BBB breakdown in subjects with chronic artery stenosis is reversible with postoperative recovery of cerebral perfusion.

The BBB constitutes an important component of the cerebral microvasculature that regulates the exchange of molecules between the blood space and the parenchyma. In the healthy brain parenchyma, the intact BBB is impermeable for large molecules such as iodinated contrast agent. In pathologic states such as neoplastic, inflammatory/infectious diseases, ischaemia, and neurodegenerative disorders, the BBB is compromised, and the diffusion of fluid, blood or contrast molecules into the extravascular space is enhanced [12,13,14]. At the molecular level, the BBB is composed of the neurovascular unit, comprising tight junctions between astrocytes and vascular endothelial cells. Permeability is related to the diffusion coefficient of the contrast agent in the capillary endothelium, which is generally assumed to be filled with water.

Computed tomography perfusion (CTP) is widely used to evaluate the BBB in vivo, with different mathematical models adapted to calculate physiological information from raw data as reviewed by Avsenik et al. [14]. Permeability surface area-product (PS) acquired using CTP measures the permeability of the BBB to contrast material [11,14,15,16,17,18]. Apart from PS CTP allows for precise quantification of such perfusion parameters as cerebral blood flow (CBF), cerebral blood volume (CBV), mean transit time (MTT), and time to peak (TTP).

In the population studied herein, the duration of internal carotid artery stenosis exceeded 5 years. Therefore, it is likely that all patients had well-developed collateral flow [19,20]. As a result, we expected deteriorated but balanced cerebral circulation parameters and increased BBB permeability (as measured by PS) in both ipsi- and contralateral sides to stenosis. Numerous experimental studies have demonstrated that effective therapy might restore, at least partially, BBB functionality [21,22]. The aim of the study was to assess the short-term effect of carotid artery stenting on BBB permeability in subjects with chronic carotid artery stenosis. We hypothesized that carotid artery stenting might diminish BBB permeability. Furthermore, we hypothesized that decline in BBB permeability would be similar in both hemispheres regardless of the side of the stenosis due to well-developed collateral blood flow (through the Willis circle) associated with generalized hypoxia and chronic inflammation. We demonstrated that PS significantly diminishes after carotid artery stenting. Therefore, we have proved that augmented BBB permeability is manageable by surgical intervention in humans*.*

## 2. Results

### 2.1. Inter-Rater Variability

Inter-rater variability for absolute values separated for both hemispheres is shown in Table S1. The inter-rater variability presents the bias (mean of differences), lower and upper agreement limits designated according to the principle of Bland and Altman [23]. Additionally two agreement coefficient were calculated, first: standard deviation of the relative differences (rSDD) obtained by subtraction each pair of observations and division by their mean, second: intra-class correlation coefficients model ICC (2.1) where each subject is measured by each rater, and reliability is calculated from a single measurement. As no systematic difference was found, data from each hemisphere were pooled together for further analysis. The data that was eventually used for analysis was the mean of CT values received from two independent raters.

### 2.2. Computed Tomography Parameters before Stenting

In all baseline cases, the CBF and CBV values were low, while MTT and TTP were high on both the ipsi- and contralateral sides compared to reference values. PS was approximately twice the normal value. Analysis of individual slices revealed that CBF was higher (+6.14%), while MTT was lower (−9.34%) on the contralateral than on the ipsilateral side (Table 1). Such a relationship was not evident in the averaged, per patient analysis (Table 2).

### 2.3. Computed Tomography Parameters after Stenting

**Figure 1:**
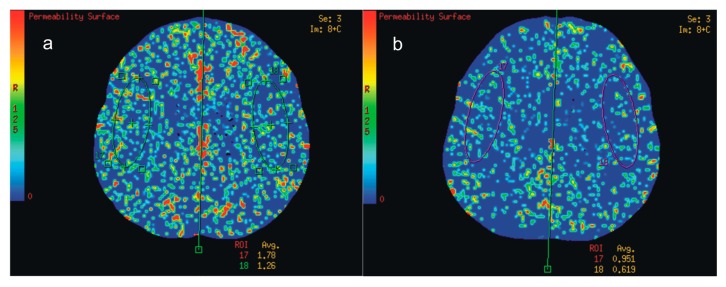
CT (Computed tomography) perfusion colour maps. Permeability surface product area (PS). Regions of interest (ROIs) placed on MCA (Middle cerebral artery) territories. (**a**) Before right internal carotid artery surgery and (**b**) after surgery. Decline in average PS after surgery was visible in both hemispheres. Regions of interest (ROIs) placed on middle cerebral artery territories (green and violet ovals on images).

All perfusion parameters improved after stenting on both the ipsilateral (CBF +22.66%, CBV +18.98%, MTT −16.09%, TTP −7.62%) and contralateral (CBF +22.27%, CBV +19.72%, MTT −14.65%, TTP −7.46%) sides. PS decreased almost by half: ipsilateral −48.11%, contralateral −45.19% (Figure 1a,b). Analysis of individual slices (Table 3) averaged per patient (Table 4) showed the same results.

Since changes were similar on the ipsi- and contralateral sides we did not observe any changes in relative values (ipsi/contra) before and after stenting (Table 5).

### 2.4. Subgroup Analysis

We performed analysis of two almost identically sized subgroups: 70–89% (21 subjects) and 90–99% (19 subjects) stenosis. Since there were no significant differences between these groups in response to surgery, we provide this analysis in supplemental files (Table S2 for all-slices analysis, Table S3 for averaged per patient analysis, Tables S4 and S5 for absolute CT perfusion parameters (mean per patient) before and after stenting).

### 2.5. Correlation and Regression Analysis

Correlation and regression analysis did not reveal any relationship between perfusion parameters (CBF, CBV, MTT, and TTP) and PS values (data not shown).

## 3. Discussion

There are two main findings of this study: (1) internal carotid artery stenting diminishes the increased BBB permeability in subjects with chronic artery stenosis, (2) such decline is similar in both the ipsi- and contralateral hemispheres. 

From the pathophysiological perspective, subjects with reduced CBF and cerebrovascular reactivity may include two different conditions: misery perfusion (or stage II ischaemia) attributable to haemodynamic compromise, and matched deteriorated metabolism attributable to incomplete infarction [24]. Having said this, under hypoxic conditions, the non-infarcted tissue is present as a consequence of a combination of reduced perfusion and moderately reduced oxygen metabolism [3]. The CBF value of 38 mL per 100 g wet weight of brain tissue per minute in subjects in Saura’s study was similar to the values reported by us. The subjects investigated in our study seemed to be very close to blood supply breakdown leading to stroke. Nevertheless, they did not cross this line and presented relatively well-compensated perfusion, although at the very low end of the acceptable range. CBV on the verge of the stroke threshold [25], long-lasting hypoxia and associated chronic inflammation are followed by reperfusion. Therefore, our results diverge from extensive available data demonstrating increased BBB permeability in the course of acute stroke [18,26,27,28,29].

The pre-surgery PS values reported in our study are below the value of 2.48 mL per 100 g wet weight of brain tissue per minute observed by Dankbaar in penumbra [28]. They are, however, similar to the values reported in the Dutch Acute Stroke study of 149 subjects with acute stroke in which most scans were performed within 5 h of symptom onset [29]. Horsch et al. did not observe any significant differences in PS between ipsi- and contralateral sides to the stroke area, as demonstrated in our study. Actually, PS in the Horsch et al. study could have been elevated before the stroke onset. Such reasoning is supported by Yang et al. [9], who found that relative ipsi/contralateral changes occur at day 7 after stroke onset and are absent on admission. From a clinical perspective, it is of critical importance to reverse the increase in BBB permeability before stroke onset. To the best of our knowledge, we prove here for the first time that augmented BBB permeability is manageable by surgical intervention in humans.

In our study, basic cerebral perfusion parameters such as CBF and MTT improved after internal carotid artery stenting on both the ipsi- and contralateral sides. Therefore, the presented results are in line with several previous reports [30,31,32,33,34]. Contralateral CBF was slightly higher and MTT lower in the all-slices analysis, tentatively suggesting better preserved circulation on the contralateral side. The contralateral artery remains the main route for collateral flow, as the reactivity of the posterior cerebral artery is much lower in subjects with chronic internal carotid artery stenosis [35]. In most studies, CBV after carotid artery stenting did not change [36,37,38]. There is, however, one report indicating CBV elevation [39]. Our study supports CBV increase. Some authors postulate that between-hemispheric relative values should be used rather than direct CBV comparisons. Merckel et al. [40] described rCBV decrease after surgery. Nevertheless, the majority of studies, in line with our results, indicate that there is no change in rCBV parameter [34,38,41]. Surgery on the ipsilateral side limits the need for collateral supply, also improving circulation in the contralateral hemisphere. Taken together, it is not surprising that our relative indices (rCBF, rCBV, rMTT, rTTP) did not change. Lack of any interdependence between perfusion parameters (CBF, CBV, MTT, and TTP) and PS underlies the multifactorial nature of BBB breakdown encompassing deteriorated perfusion and an extensive inflammatory state [42].

All subjects had had a stenosis diagnosed by a Doppler examination more than 5 years before surgery. Owing to strict inclusion criteria consisting of stenosis of <70% within a single ICA with concomitant neurological symptoms (dizziness, tinnitus, visual disturbances and headaches), our population was very homogeneous in terms of cerebral haemodynamic parameters and was characterized by low CBF and low CBV [43]. As a result, in all patients in the studied population, stenosis on the contralateral side was haemodynamically not significant (<50%) [44,45,46,47]. Although the relatively narrow population can be seen as a limitation of this study, population homogeneity increased the reliability of our results. Patients with bilateral haemodynamically significant (i.e., >70%) stenosis of cranial arteries were excluded from the study.

Carotid angioplasty and stenting for the treatment of severe carotid obstructive disease is becoming more widely performed and is now widely accepted as a less invasive technique that provides an attractive alternative for many patients. Complication rates range from 0.9% to 9.3% following the experience of different centres [48,49,50,51,52,53]. The potential procedural and periprocedural complications that may be related to carotid angioplasty and stenting may be divided on two types of complications: 1) minor complications (carotid artery spasm, sustained hypotension/bradycardia, carotid artery dissection, contrast encephalopathy, TIAs, complications at the site of the vascular access), 2) major complications (embolic stroke, intracranial haemorrhage, cerebral hyperperfusion syndrome, carotid perforation, acute stent thrombosis). During our studies, we did not observe any significant side effects of the procedure. Only one patient had TIA episodes, and four had minor complications at the site of the vascular access (such as the bruises).

Quantitative CTP data is highly dependent on the post-processing software. Software differences are frequently considered the main cause of variability in perfusion results relative to interoperator and intraoperator differences [54,55,56]. We used equipment manufactured by one producer and used the same post-processing procedure for all subjects, increasing the reliability of the study. Furthermore, both experienced neuroradiologists independently drew two standardized elliptical mirrored ROIs (an area of approximately 10 cm^2^ each) on the section level of the reference CT image (Figure 1a,b) over the cortical grey matter of the middle cerebral artery territory. The study sample size was based on prospective test power analysis (Figure S1). Finally, in all subjects we used a coverage size of 40 mm, which is well-suited for detecting perfusion parameters owing to the high density of scans [43].

We used a total scan time of 45 s. Longer acquisition time during CTP examination is more favourable for measurement of PS to avoid overestimation. For the first-pass CTP data, it is generally recommended to obtain for at least 90 s and it should be combined with delay correction for 90-s CTP acquisition. According to Patlak model shorter acquisitions may lead to overestimated BBB values [18,57]. Thus, the PS values we observed may be overestimated. Nevertheless, even if the results are overestimated, the same procedure was used before and after stenting. Therefore, the direction and significance of changes are not undermined. Importantly, in Johnson and Wilson model (this technique has been used in our investigations) PS and CBV are independent parameters. MTT, CBV, CBF are calculated with the first phase of impulse residue function (IRF) and PS is calculated with the second phase of IRF [58]. Therefore, PS was not influenced by elevated CBV.

CTP in most cases remains the first choice in the management of patients with acute stroke and other cerebrovascular disorders. It is widely available and provides an easy and fast way of obtaining both morphological and perfusion-related parameters. The main drawback of CTP are concerns raised over high radiation dose. In our study, a conventional angiography was the necessary part of carotid artery stenting to confirm the degree of stenosis. However, a new method to create an ultra-low-dose virtual perfusion computer tomography by enhancing it with computed tomography angiography information seems to be under way [59]. Computed tomography angiography allows for almost isotropic resolution of the images and combined with PS parameter obtained from CTP may potentially deliver unique information about human brain pathophysiology.

Dynamic contrast-enhanced (DCE) magnetic resonance imaging (MRI) with/without pre- and post-contrast fluid attenuation inversion recovery (FLAIR) imaging has several advantages in terms of BBB assessments compared to CTP. In particular, DCE-MRI is not associated with radiation and can be linked with blood oxygen level dependent MRI to provide functional information on the effect of prolonged ischemia and consequent hypoxia on central nervous system. The main disadvantage of DCE-MRI is that the information it provides with respect to BBB values is very relative in nature. Nevertheless, further research on BBB permeability with both methods is planned by our team. We aim at BBB measurement with modified CTP two-phase, low radiation dose protocol and DCE-MRI combined with the BOLD-MRI methodology.

## 4. Material and Methods

### 4.1. Patient Population

During 2010–2014, 40 patients (17 females (42.5%) and 23 males (57.5%); mean age of 70.20 ± SD 7.51 years) underwent an internal carotid artery stenting procedure in the Department of Cardiac and Vascular Surgery of the Medical University of Gdansk. The experimental protocol and the study were approved by the relevant ethics committee of the Medical University of Gdansk (NKBBN:383/2008 dated 10 March 2009). All volunteers gave written informed consent to participate in the study.

Patients with stenosis of more than 70% within a single internal carotid artery with neurological symptoms such as transient ischaemic attack (TIA), transient monocular blindness, noncardiogenic fainting, unconsciousness, minor ischemic stroke (e.g., lacunar), dizziness, tinnitus, visual disturbances, and headaches were included in the study (Table 6). Symptomatic carotid disease was defined as focal neurologic symptoms that were sudden in onset and referable to the appropriate carotid artery distribution (ipsilateral to significant carotid atherosclerotic pathology), including one or more transient ischemic attacks characterized by focal neurologic dysfunction or transient blindness, or one or more minor (nondisabling) ischemic strokes [4]. The definition is contingent on the occurrence of carotid symptoms within the previous six months [4,60].

Based on Doppler ultrasound diagnosis, in all subjects, the duration of carotid artery stenosis exceeded 5 years. A general examination was performed prior to the experiment; subjects with an abnormal blood pressure, haematocrit ratio or heart rate values were excluded from the study. Subjects with bilateral haemodynamically significant (i.e., more than 70%) stenosis of internal carotid arteries, complete occlusion of one vessel or subjects who had experienced broad ischaemic brain stroke were excluded from the study. Subjects with renal insufficiency, uncontrolled hyperthyroidism, and hypersensitivity to iodine or history of adverse effects following the administration of contrast agents were also excluded from the study.

### 4.2. Angiography

Confirmatory digital subtractive angiography was performed prior to carotid artery stenting. Patients were considered for this study when the internal carotid artery stenosis exceeded 70%, and the stenosis on the contralateral side was less than 50% (using North American Symptomatic Carotid Endarterectomy Trial method) [4]. Selective angiography of the target vessel was then performed to reveal the size of the carotid lesion, degree of stenosis, morphology of the internal carotid artery and its contribution to the circle of Willis. Only patients with a complete circle of Willis and normal vertebral arteries were included in this study.

### 4.3. Experimental Design

The main aim of this study was to prospectively investigate how PS changes after carotid stenting. Therefore, the sample size was based only on calculations for the PS and *t*-test for dependent samples. Earlier observations revealed a mean value and standard deviation before stenting of 1.5 ± 0.8, and an expected effect size of 0.5 with a standard deviation of PS after stenting as half of the value before surgery. The minimum sample size for Alfa = 0.05 and 1-Beta = 0.9 was 36 (Figure S1). However, the authors decided to randomize 40 patients from the population using a simple draw without replacement. A population size of 40 was essential not only because of this satisfied previous assumptions (minimum sample size) but also because it allowed (thanks to the Central Limit Theorem) the use of parametric tests, despite the lack of normality.

### 4.4. Imaging Protocol

All non-contrast CTs (NCCT) and CTPs were performed on a 64-MDct Light Speed VCT XT scanner (GE Healthcare Technologies, Waukesha, WI, USA).

To objectively assess the cerebral blood supply in all patients, two CTP examinations were carried out: the first was performed 24 h before the internal carotid artery stenting procedure while the second was performed 6–8 weeks after stent implantation.

The NCCT (with 5-mm contiguous axial sections from vertex to skull base) used imaging parameters of 140 kVp, 335 mAs, 0.9 s rotation time, the number of images = 56, total exposure time = 6.3 s, and CDTIvol between 50 and 60 mGy.

All CTPs were obtained from 40 mm thick axial sections. Similar to the previous studies [26,61], the first scan was set at the basal ganglia, above the level of the circle of Willis. The gantry angle was parallel to the orbitomeatal line to prevent incorporation of the eye lenses. A cine mode series was performed beginning 5 s after the intravenous administration of 40 mL of iodinated contrast (Optiray 350 Mallinckrodt, St. Louis, MO, USA) at 4 mL/s by a power injector into an antecubital vein. After contrast injection, an additional bolus of 40 mL of physiological saline was administered.

The CTP imaging parameters were 80 kVp, 150 mAs, slice thickness = 5 mm, rotation time = 1/s, number of images per rotation = 8, cine time between images = 0.5 s, image matrix = 512 × 512, field-of-view (FOV) = 25 cm, time interval between reconstructed images = 0.5 s, interval = 0 mm. A total of 360 slices was obtained with a total scan time of 45 s. CDTIvol was approximately 390 mGy per examination.

All acquisition parameters were applied as instructed by the manufacturer for the brain perfusion study. The arterial input function was chosen within the anterior cerebral artery. The venous output region was selected from the superior sagittal sinus.

### 4.5. Image Post-Processing

The raw datasets were loaded onto a dedicated diagnostic workstation (AW 4 GE Healthcare Technologies, Milwaukee, WI, USA) equipped with a professional postprocessing software package to generate colour overlay maps of dynamic cerebral enhancement data (CT Perfusion version 4 (v 4.3.1), GE Healthcare Technologies, Milwaukee, WI, USA).

This application offers two different evaluation modes. The first mode, the “neuro brain stroke” mode, calculates the perfusion metrics of MTT, CBF, CBV, and TTP for brain stroke assessment using the maximal slope method [32,62].

MTT characterizes the average time of contrast agent residence within the tissue. In mathematical terms, the mean transit time is computed as the time between the initial impulse and the time of arrival. MTT is computed and displayed in seconds.

CBF is derived from the initial value of the impulse residue function and is computed and displayed in mL per 100 g of wet tissue per minute.

CBV is computed and displayed in mL per 100 g of wet tissue. The blood volume is the product of the blood flow and the mean transit time: CBV = CBF × MTT.

TTP is the time between the onset of the enhancement transient (last pre-enhancement image) and the peak value of the time curve (image with the maximum value before the first post-enhancement image). Time-to-peak is computed and displayed in seconds, using the raw time curve data directly.

The second mode, known as the “neuro brain tumour” mode, calculates microvascular permeability (PS) and fractional blood volume based on the Johnson and Wilson model [16,17].

PS is computed and displayed in mL per 100 g of wet tissue per minute. It is computed from the impulse residue function. Contrast agent diffusion appears in the impulse residue function as a residual enhancement that occurs after the initial impulse response and decreases exponentially with time.

Blind to clinical information (medical history, side and time of operation), two experienced neuroradiologists independently drew two standardized elliptical mirrored regions of interest (ROIs) manually. Each ROI (approximately 10 cm^2^ each) was determined on the all analysed levels (Figure 1a,b), over the cortical grey matter centred 20 mm from the edge of the brain. The large vessels were automatically excluded via brain perfusion software.

The absolute values of CT perfusion parameters (MTT, TTP, CBF, CBV and PS) of one hemisphere in the region of middle cerebral artery distribution and contralateral mirroring areas in functional maps were measured.

### 4.6. Statistical Analysis

To compare results before and after stenting, differences in absolute CT values (MTT, CBV, CBF, MTT, PS) and relative values (rMTT, rCBV, rCBF, rTTP, rPS—obtained as the ratio of appropriate values from ipsilateral side to contralateral side to stenosis) were analysed.

Data from all individual slices (rather than averaged per patient *C*_t_ values) were used for analysis. Although the mean values were the same in both approaches, the use of all slices seemed to be more appropriate for two main reasons: sample sizes were greater and standard deviations were more correct for the whole brain. Results from both comparisons are presented and discussed.

Normality was verified using the W Shapiro–Wilk test. Differences between mean values in small groups were examined by Welch’s *t*-test (symbol *t*) or Mann-Whitney *U* test (symbol *z*) if necessary.

Data from large groups did not need to satisfy the normality assumption for the use of the parametric *t*-test, according to the Central Limit Theorem. Hence, differences in absolute and relative values between the perfusion methods were assessed using only the parametric *t*-test.

The level of significance was set at α = 0.05. All calculated *p*-values were for two-tailed tests. All raw data were analysed using the statistical software Statistica 10 (StatSoft, Tulsa, OK, USA).

## 5. Conclusions

To conclude, we analysed a relatively homogeneous population with chronic carotid artery stenosis, low CBF, and low CBV. Before surgery, BBB permeability was elevated due to long-lasting brain hypoxia, oxidative stress, and inflammation, and the perfusion parameters were likely close to the stroke threshold. We have shown that internal carotid artery stenting diminishes BBB permeability in such subjects. The decline in BBB permeability was symmetrical on the ipsi- and contralateral sides to the stenosis. To the best of our knowledge, we have proved for the first time that augmented BBB permeability is manageable by surgical intervention in humans. Further studies in subjects with carotid artery stenosis are warranted.

## Figures and Tables

**Table 1:** Absolute CT perfusion parameter values before stenting (all slices included). Cerebral blood flow (CBF) and mean transit time (MTT) values revealed that the contralateral side was slightly better perfused.

Brain Hemisphere	*t*-Test for Independent Samples							
	CT	Mean Ipsi	Mean Contra	*t*	No.	*p*-Value	SD Ipsi	SD Contra
Stented side–before vs. Contralateral side–before	CBF	37.619	39.927	−2.716	320	0.007	11.302	10.166
Stented side–before vs. Contralateral side–before	CBV	1.771	1.765	0.232	320	0.817	0.374	0.351
Stented side–before vs. Contralateral side–before	MTT	4.730	4.288	4.490	320	0.000	1.470	0.974
Stented side–before vs. Contralateral side–before	TTP	26.088	25.758	1.220	320	0.223	3.401	3.434
Stented side–before vs. Contralateral side–before	PS	1.617	1.498	1.890	320	0.059	0.803	0.787

CBF—Cerebral Blood Flow, CBV—Cerebral Blood Volume, MTT—Mean Transit Time, TTP—Time to Peak, PS—Permeability Surface Area-Product, SD—Standard Deviation.

**Table 2:** Absolute CT perfusion parameter values before stenting (mean per patient). In the averaged, per patient analysis there were no differences between ipsi- and contra-lateral sides to the stenosis.

Brain Hemisphere	*t*-Test for Independent Samples							
	CT	Mean Ipsi	Mean Contra	*t*	No.	*p*-Value	SD Ipsi	SD Contra
Stented side–before vs. Contralateral side–before	CBF	37.619	39.927	−1.295	40	0.199	8.807	7.034
Stented side–before vs. Contralateral side–before	CBV	1.771	1.765	0.106	40	0.916	0.291	0.272
Stented side–before vs. Contralateral side–before	MTT	4.730	4.288	1.751	40	0.085	1.368	0.827
Stented side–before vs. Contralateral side–before	TTP	26.088	25.758	0.434	40	0.665	3.391	3.403
Stented side–before vs. Contralateral side–before	PS	1.617	1.498	0.746	40	0.458	0.717	0.707

CBF—Cerebral Blood Flow, CBV—Cerebral Blood Volume, MTT—Mean Transit Time, TTP—Time to Peak, PS—Permeability Surface Area-Product, SD—Standard Deviation.

**Table 3:** Absolute CT perfusion parameter values (all slices included) before and after stenting. In both hemispheres increases in CBF and cerebral blood volume (CBV) were observed while MTT and time to peak (TTP) diminished.

Variable	*t*-Test for Dependent Samples					
	Side	Mean	SD	No.	*t*	*p*-Value
MTT–1	Ipsilateral side	4.730	1.470	320	13.172	<0.001
MTT–2		3.969	1.160			
MTT–1	Contralateral side	4.288	0.974	320	16.158	<0.001
MTT–2		3.660	0.862			
CBV–1	Ipsilateral side	1.771	0.374	320	–13.463	<0.001
CBV–2		2.107	0.573			
CBV–1	Contralateral side	1.765	0.351	320	–14.167	<0.001
CBV–2		2.113	0.560			
CBF–1	Ipsilateral side	37.619	11.302	320	–14.845	<0.001
CBF–2		46.143	12.842			
CBF–1	Contralateral side	39.927	10.166	320	–12.551	<0.001
CBF–2		48.817	15.349			
TTP–1	Ipsilateral side	26.088	3.401	320	19.919	<0.001
TTP–2		24.100	3.162			
TTP–1	Contralateral side	25.758	3.434	320	20.623	<0.001
TTP–2		23.837	3.208			
PS–1	Ipsilateral side	1.617	0.803	320	19.259	<0.001
PS–2		0.839	0.370			
PS–1	Contralateral side	1.498	0.787	320	15.945	<0.001
PS–2		0.821	0.396			

CBF—Cerebral Blood Flow, CBV—Cerebral Blood Volume, MTT—Mean Transit Time, TTP—Time to Peak, PS—Permeability Surface Area-Product, SD—Standard Deviation.

**Table 4:** Absolute CT perfusion parameter values (mean per patient) before or after stenting. In both hemispheres increases in CBF and CBV were observed while MTT and TTP diminished.

Variable	*t*-Test for Dependent Samples					
	Side	Mean	SD	No.	*t*	*p*-Value
MTT–1	Ipsilateral side	4.730	1.368	40	5.254	<0.001
MTT–2		3.969	1.081			
MTT–1	Contralateral side	4.288	0.827	40	7.841	<0.001
MTT–2		3.660	0.754			
CBV–1	Ipsilateral side	1.771	0.291	40	–5.749	<0.001
CBV–2		2.107	0.512			
CBV–1	Contralateral side	1.765	0.272	40	–5.932	<0.001
CBV–2		2.113	0.497			
CBF–1	Ipsilateral side	37.619	8.807	40	–6.953	<0.001
CBF–2		46.143	10.003			
CBF–1	Contralateral side	39.927	7.034	40	–5.327	<0.001
CBF–2		48.817	12.798			
TTP–1	Ipsilateral side	26.088	3.391	40	7.376	<0.001
TTP–2		24.100	3.153			
TTP–1	Contralateral side	25.758	3.403	40	8.094	<0.001
TTP–2		23.837	3.194			
PS–1	Ipsilateral side	1.617	0.717	40	8.189	<0.001
PS–2		0.839	0.313			
PS–1	Contralateral side	1.498	0.707	40	6.645	<0.001
PS–2		0.821	0.311			

CBF—Cerebral Blood Flow, CBV—Cerebral Blood Volume, MTT—Mean Transit Time, TTP—Time to Peak, PS—Permeability Surface Area-Product, SD—Standard Deviation.

**Table 5:** Relative CT values (all slices included). No changes were observed after surgery.

Variable	*t*-Test for Dependent Samples					
	CT Value	Mean	SD	*n*	*t*	*p*-Value
Relative before	CBF	0.954	0.232	320	–1.620	0.106
Relative after	CBF	0.973	0.211			
Relative before	CBV	1.010	0.130	320	0.902	0.368
Relative after	CBV	1.003	0.125			
Relative before	MTT	1.124	0.322	320	1.151	0.250
Relative after	MTT	1.108	0.312			
Relative before	PS	1.192	0.649	320	1.068	0.287
Relative after	PS	1.146	0.586			
Relative before	TTP	1,014	0.049	320	0.690	0.491
Relative after	TTP	1.012	0.044			

CBF—Cerebral Blood Flow, CBV—Cerebral Blood Volume, MTT—Mean Transit Time, TTP—Time to Peak, PS—Permeability Surface Area-Product, SD—Standard Deviation.

**Table 6:** Risk factors and concomitant diseases in the studied population.

Concomitant Disease	Number of Cases
Smokers	15
Hypertension	34
Diabetes mellitus type 2	11
Transient ischemic attack	10
Stroke	5
Coronary artery disease	24
Lipid disorders	11
Myocardial infarction	14

## Supplementary Materials

Supplementary materials can be found at www.mdpi.com/1422-0067/18/5/1008/s1.
