# Supplementary material for: Carotid Artery Stenting and Blood–Brain Barrier Permeability in Subjects with Chronic Carotid Artery Stenosis
Source: Int J Mol Sci. 2017 May 8;18(5):1008. doi: 10.3390/ijms18051008 (PMC5454921; doi:10.3390/ijms18051008)
Supplement: Supplementary file 1 [file ijms-18-01008-s001.pdf]

# Carotid Artery Stenting and Blood–Brain Barrier Permeability in Subjects with Chronic Carotid Artery Stenosis

Arkadiusz Szarmach, Grzegorz Halena, Mariusz Kaszubowski, Maciej Piskunowicz, Michal Studniarek, Piotr Lass, Edyta Szurowska and Pawel J. Winklewski

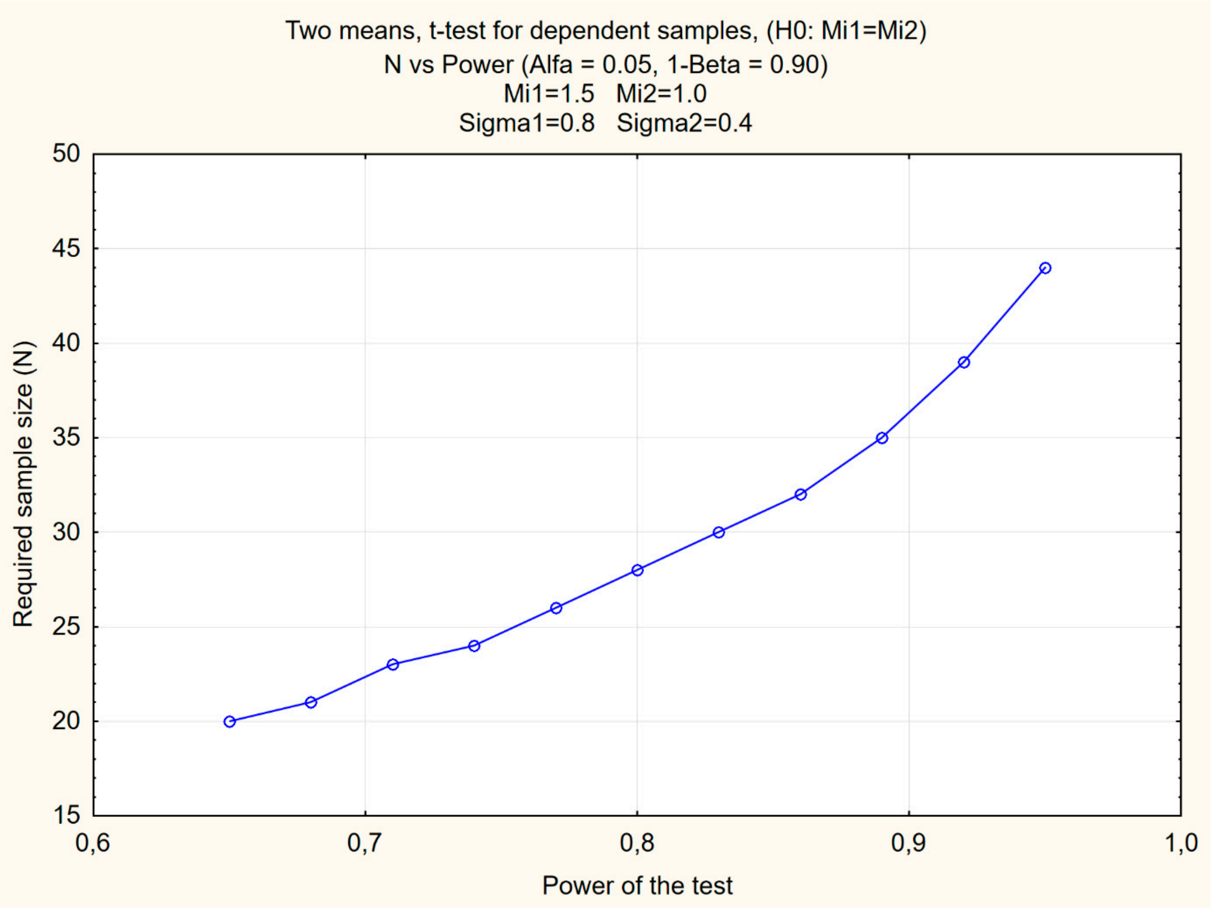


Figure S1. The sample size calculation. The minimum sample size for Alfa=0.05 and 1-Beta=0.9 is equal to 36 subjects.

Table S1. Inter-observer variability before (1) and after (2) surgery. The inter-rater variability presents the bias (mean of differences), lower and upper agreement limits designated according to the principle of Bland and Altman [23] and the standard deviation of the relative differences calculated as the difference between each pair of observations divided by their mean.

| CT | Side | Bias | SD | Lower Agreement Limit | Upper Agreement Limit | rSDD | ICC(2,1) |
|---|---|---|---|---|---|---|---|
| **MTT – 1** | Ipsilateral side | 0.003 | 0.257 | -0.502 | 0.508 | 6.16% | 0.985 |
| **MTT – 1** | Contralateral side | -0,027 | 0.203 | -0.425 | 0.409 | 5.05% | 0.978 |
| **MTT – 2** | Ipsilateral side | -0.008 | 0.180 | -0.361 | 0.345 | 4.96% | 0.988 |
| **MTT – 2** | Contralateral side | 0.001 | 0.182 | -0.354 | 0.356 | 5.36% | 0.978 |
| **CBV – 1** | Ipsilateral side | -0.004 | 0.122 | -0.244 | 0.235 | 7.12% | 0.948 |
| **CBV – 1** | Contralateral side | -0.005 | 0.121 | -0.242 | 0.232 | 6.90% | 0.942 |
| **CBV – 2** | Ipsilateral side | -0.003 | 0.100 | -0.199 | 0.194 | 5.18% | 0.985 |
| **CBV – 2** | Contralateral side | -0.011 | 0.107 | -0.220 | 0.199 | 5.55% | 0.982 |
| **CBF – 1** | Ipsilateral side | 0.150 | 1.260 | -2.321 | 2.621 | 4.07% | 0.994 |
| **CBF – 1** | Contralateral side | -0.440 | 1.050 | -2.498 | 1.818 | 2.74% | 0.993 |
| **CBF – 2** | Ipsilateral side | -0.187 | 1.149 | -2.439 | 2.065 | 2.81% | 0.996 |
| **CBF – 2** | Contralateral side | -0.205 | 1.306 | -2.766 | 2.355 | 2.75% | 0.996 |
| **TTP – 1** | Ipsilateral side | -0.306 | 0.676 | -1.632 | 1.019 | 2.56% | 0.977 |
| **TTP – 1** | Contralateral side | -0.157 | 0.351 | -0.846 | 0.531 | 1.42% | 0.994 |
| **TTP – 2** | Ipsilateral side | -0.133 | 0.502 | -1.116 | 0.851 | 2.05% | 0.987 |
| **TTP – 2** | Contralateral side | -0.154 | 0.480 | -1.094 | 0.786 | 2.01% | 0.988 |
| **PS – 1** | Ipsilateral side | -0.006 | 0.086 | -0.175 | 0.163 | 6.67% | 0.994 |
| **PS – 1** | Contralateral side | -0.013 | 0.078 | -0.166 | 0.140 | 6.48% | 0.995 |
| **PS – 2** | Ipsilateral side | 0.011 | 0.061 | -0.109 | 0.131 | 10.16% | 0.986 |
| **PS – 2** | Contralateral side | 0.003 | 0.071 | -0.136 | 0.143 | 11.38% | 0.984 |

SDtd. Dev. – standard deviation, rSDD - standard deviation of the relative differences, ICC(2.1) - intra-class correlation coefficient.

Table S2. Subgroup analysis for absolute CT perfusion parameter values (all slices) in two subgroups: 70–89% (21 subjects) and 90–99% (19 subjects) stenosis before (1) and after (2) stenting. Both groups benefited from the surgery.

| Variable | t-test for dependent samples | | | | | | |
|---|---|---|---|---|---|---|---|
| | **Side** | **Stenosis** | **Mean** | **SD** | **No.** | **t** | **p-value** |
| **MTT – 1** | Ipsilateral side | 70-89% | 4.906 | 1.699 | 168 | 9.999 | <0.001 |
| **MTT – 2** | | | 3.962 | 1.213 | | | |
| **MTT – 1** | Ipsilateral side | 90-99% | 4.536 | 1.141 | 152 | 9.525 | <0.001 |
| **MTT – 2** | | | 3.976 | 1.103 | | | |
| **MTT – 1** | Contralateral side | 70-89% | 4.444 | 1.081 | 168 | 11.768 | <0.001 |
| **MTT – 2** | | | 3.759 | 0.890 | | | |
| **MTT – 1** | Contralateral side | 90-99% | 4.115 | 0.809 | 152 | 11.251 | <0.001 |
| **MTT – 2** | | | 3.551 | 0.820 | | | |
| **CBV – 1** | Ipsilateral side | 70-89% | 1.738 | 0.326 | 168 | -9.024 | <0.001 |
| **CBV – 2** | | | 2.026 | 0.460 | | | |
| **CBV – 1** | Ipsilateral side | 90-99% | 1.810 | 0.418 | 152 | -10.074 | <0.001 |
| **CBV – 2** | | | 2.196 | 0.666 | | | |
| **CBV – 1** | Contralateral side | 70-89% | 1.723 | 0.313 | 168 | -10.607 | <0.001 |
| **CBV – 2** | | | 2.038 | 0.435 | | | |
| **CBV – 1** | Contralateral side | 90-99% | 1.811 | 0.384 | 152 | -9.644 | <0.001 |
| **CBV – 2** | | | 2.195 | 0.664 | | | |
| **CBF – 1** | Ipsilateral side | 70-89% | 36.836 | 10.913 | 168 | -9.666 | <0.001 |
| **CBF – 2** | | | 44.266 | 12.590 | | | |
| **CBF – 1** | Ipsilateral side | 90-99% | 38.486 | 11.693 | 152 | -11.426 | <0.001 |
| **CBF – 2** | | | 48.217 | 12.840 | | | |
| **CBF – 1** | Contralateral side | 70-89% | 38.396 | 8.142 | 168 | -8.188 | <0.001 |
| **CBF – 2** | | | 45.121 | 12.246 | | | |
| **CBF – 1** | Contralateral side | 90-99% | 41.619 | 11.811 | 152 | -9.766 | <0.001 |
| **CBF – 2** | | | 52.904 | 17.315 | | | |
| **TTP – 1** | Ipsilateral side | 70-89% | 26.185 | 2.594 | 168 | 17.899 | <0.001 |
| **TTP – 2** | | | 24.002 | 2.219 | | | |
| **TTP – 1** | Ipsilateral side | 90-99% | 25.982 | 4.119 | 152 | 11.093 | <0.001 |
| **TTP – 2** | | | 24.209 | 3.956 | | | |
| **TTP – 1** | Contralateral side | 70-89% | 25.939 | 2.527 | 168 | 16.328 | <0.001 |
| **TTP – 2** | | | 23.905 | 2.128 | | | |
| **TTP – 1** | Contralateral side | 90-99% | 25.559 | 4.217 | 152 | 12.888 | <0.001 |
| **TTP – 2** | | | 23.762 | 4.090 | | | |
| **PS – 1** | Ipsilateral side | 70-89% | 1.665 | 0.748 | 168 | 14.841 | <0.001 |
| **PS – 2** | | | 0.912 | 0.358 | | | |
| **PS – 1** | Ipsilateral side | 90-99% | 1.564 | 0.858 | 152 | 12.578 | <0.001 |
| **PS – 2** | | | 0.759 | 0.366 | | | |
| **PS – 1** | Contralateral side | 70-89% | 1.572 | 0.749 | 168 | 14.552 | <0.001 |
| **PS – 2** | | | 0.888 | 0.358 | | | |
| **PS – 1** | Contralateral side | 90-99% | 1.417 | 0.823 | 152 | 9.188 | <0.001 |
| **PS – 2** | | | 0.746 | 0.423 | | | |

CBF- Cerebral Blood Flow, CBV- Cerebral Blood Volume, MTT- Mean Transit Time, TTP- Time to Peak, PS- Permeability Surface Area-Product , SD- Standard Deviation.

Table S3. Subgroup analysis for absolute CT perfusion parameter values (mean per patient) in two subgroups: 70–89% (21 subjects) and 90–99% (19 subjects) stenosis before (1) and after (2) surgery. Both groups benefited from the surgery.

| **Variable** | | | **Tests for dependent samples** | | | | |
|---|---|---|---|---|---|---|---|
| | **Side** | **Stenosis** | **Mean** | **SD** | **N** | **t/z** | **p-value** |
| **MTT – 1** | Ipsilateral side | 70-89% | 4.906 | 1.616 | 21 | 4.015 | <0.001 |
| **MTT – 2** | | | 3.962 | 1.142 | | | |
| **MTT – 1** | Ipsilateral side | 90-99% | 4.536 | 1.039 | 19 | 4.320 | <0.001 |
| **MTT – 2** | | | 3.976 | 1.041 | | | |
| **MTT – 1** | Contralateral side | 70-89% | 4.444 | 0.954 | 21 | 4.015 | <0.001 |
| **MTT – 2** | | | 3.759 | 0.787 | | | |
| **MTT – 1** | Contralateral side | 90-99% | 4.115 | 0.642 | 19 | 3.823 | <0.001 |
| **MTT – 2** | | | 3.551 | 0.721 | | | |
| **CBV – 1** | Ipsilateral side | 70-89% | 1.737 | 0.216 | 21 | 4.015 | <0.001 |
| **CBV – 2** | | | 2.026 | 0.381 | | | |
| **CBV – 1** | Ipsilateral side | 90-99% | 1.810 | 0.359 | 19 | 3.743 | <0.001 |
| **CBV – 2** | | | 2.196 | 0.624 | | | |
| **CBV – 1** | Contralateral side | 70-89% | 1.723 | 0.221 | 21 | 3.945 | <0.001 |
| **CBV – 2** | | | 2.038 | 0.360 | | | |
| **CBV – 1** | Contralateral side | 90-99% | 1.811 | 0.318 | 19 | 3.783 | <0.001 |
| **CBV – 2** | | | 2.195 | 0.615 | | | |
| **CBF – 1** | Ipsilateral side | 70-89% | 36.836 | 8.360 | 21 | 4.015 | <0.001 |
| **CBF – 2** | | | 44.266 | 9.416 | | | |
| **CBF – 1** | Ipsilateral side | 90-99% | 38.486 | 9.428 | 19 | 3.823 | <0.001 |
| **CBF – 2** | | | 48.217 | 10.471 | | | |
| **CBF – 1** | Contralateral side | 70-89% | 38.396 | 4.356 | 21 | 3.980 | <0.001 |
| **CBF – 2** | | | 45.121 | 9.050 | | | |
| **CBF – 1** | Contralateral side | 90-99% | 41.619 | 8.965 | 19 | 3.783 | <0.001 |
| **CBF – 2** | | | 52.904 | 15.175 | | | |
| **TTP – 1** | Ipsilateral side | 70-89% | 26.185 | 2.595 | 21 | 4.015 | <0.001 |
| **TTP – 2** | | | 24.002 | 2.213 | | | |
| **TTP – 1** | Ipsilateral side | 90-99% | 25.982 | 4.172 | 19 | 4.012 | <0.001 |
| **TTP – 2** | | | 24.209 | 4.009 | | | |
| **TTP – 1** | Contralateral side | 70-89% | 25.939 | 2.443 | 21 | 4.015 | <0.001 |
| **TTP – 2** | | | 23.905 | 2.102 | | | |
| **TTP – 1** | Contralateral side | 90-99% | 25.559 | 4.287 | 19 | 3.823 | <0.001 |
| **TTP – 2** | | | 23.762 | 4.145 | | | |
| **PS – 1** | Ipsilateral side | 70-89% | 1.665 | 0.678 | 21 | 6.366 | <0.001 |
| **PS – 2** | | | 0.912 | 0.302 | | | |
| **PS – 1** | Ipsilateral side | 90-99% | 1.564 | 0.751 | 19 | 3.823 | <0.001 |
| **PS – 2** | | | 0.759 | 0.313 | | | |
| **PS – 1** | Contralateral side | 70-89% | 1.572 | 0.675 | 21 | 6.098 | <0.001 |
| **PS – 2** | | | 0.888 | 0.292 | | | |
| **PS – 1** | Contralateral side | 90-99% | 1.417 | 0.751 | 19 | 3.501 | <0.001 |
| **PS – 2** | | | 0.746 | 0.322 | | | |

CBF- Cerebral Blood Flow, CBV- Cerebral Blood Volume, MTT- Mean Transit Time, TTP- Time to Peak, PS- Permeability Surface Area-Product , SD- Standard Deviation.

Table S4. Tests for independent subgroups of absolute CT perfusion parameter (mean per patient) in two subgroups: 70–89% (21 subjects) and 90–99% (19 subjects) stenosis before stenting. There were no differences between these subgroups.

| **Stenosis** | **Test for independent samples** | | | | | | |
|---|---|---|---|---|---|---|---|
| | **Side** | **CT Value** | **Mean** | **SD** | **N** | **t** | **p-value** |
| 70-89% | Ipsilateral | CBF | 36.836 | 8.360 | 21 | -0.583 | 0.563 |
| 90-99% | Ipsilateral | CBF | 38.486 | 9.428 | 19 | | |
| 70-89% | Contralateral | CBF | 38.396 | 4.356 | 21 | -1.423 | 0.167 |
| 90-99% | Contralateral | CBF | 41.619 | 8.965 | 19 | | |
| 70-89% | Ipsilateral | CBV | 1.737 | 0.216 | 21 | -0.771 | 0.447 |
| 90-99% | Ipsilateral | CBV | 1.810 | 0.359 | 19 | | |
| 70-89% | Contralateral | CBV | 1.723 | 0.221 | 21 | -1.002 | 0.324 |
| 90-99% | Contralateral | CBV | 1.811 | 0.318 | 19 | | |
| 70-89% | Ipsilateral | MTT | 4.906 | 1.616 | 21 | 0.871 | 0.390 |
| 90-99% | Ipsilateral | MTT | 4.536 | 1.039 | 19 | | |
| 70-89% | Contralateral | MTT | 4.444 | 0.954 | 21 | 1.292 | 0.205 |
| 90-99% | Contralateral | MTT | 4.115 | 0.642 | 19 | | |
| 70-89% | Ipsilateral | PS | 1.665 | 0.678 | 21 | 0.440 | 0.663 |
| 90-99% | Ipsilateral | PS | 1.564 | 0.773 | 19 | | |
| 70-89% | Contralateral | PS | 1.572 | 0.675 | 21 | 0.682 | 0.500 |
| 90-99% | Contralateral | PS | 1.417 | 0.751 | 19 | | |
| 70-89% | Ipsilateral | TTP | 26.185 | 2.595 | 21 | 0.182 | 0.857 |
| 90-99% | Ipsilateral | TTP | 25.982 | 4.172 | 19 | | |
| 70-89% | Contralateral | TTP | 25.939 | 2.443 | 21 | 0.339 | 0.737 |
| 90-99% | Contralateral | TTP | 25.559 | 4.287 | 19 | | |

CBF- Cerebral Blood Flow, CBV- Cerebral Blood Volume, MTT- Mean Transit Time, TTP- Time to Peak, PS- Permeability Surface Area-Product , SD- Standard Deviation.

Table S5. Tests for independent subgroups of absolute CT perfusion parameter (mean per patient) in two subgroups: 70–89% (21 subjects) and 90–99% (19 subjects) stenosisafter stenting. There were no differences between these subgroups.

| **Stenosis** | **Test for independent samples** | | | | | | |
|---|---|---|---|---|---|---|---|
| | **Side** | **CT Value** | **Mean** | **SD** | **N** | **t** | **p-value** |
| 70-89% | Ipsilateral | CBF | 44.266 | 9.416 | 21 | -1.250 | 0.219 |
| 90-99% | Ipsilateral | CBF | 48.217 | 10.471 | 19 | | |
| 70-89% | Contralateral | CBF | 45.121 | 9.050 | 21 | -1.944 | 0.062 |
| 90-99% | Contralateral | CBF | 41.619 | 15.175 | 19 | | |
| 70-89% | Ipsilateral | CBV | 2.026 | 0.381 | 21 | -1.031 | 0.311 |
| 90-99% | Ipsilateral | CBV | 2.196 | 0.624 | 19 | | |
| 70-89% | Contralateral | CBV | 2.038 | 0.360 | 21 | -0.971 | 0.340 |
| 90-99% | Contralateral | CBV | 2.195 | 0.615 | 19 | | |
| 70-89% | Ipsilateral | MTT | 3.962 | 1.142 | 21 | -0.040 | 0.968 |
| 90-99% | Ipsilateral | MTT | 3.976 | 1.041 | 19 | | |
| 70-89% | Contralateral | MTT | 3.759 | 0.787 | 21 | 0.871 | 0.389 |
| 90-99% | Contralateral | MTT | 3.551 | 0.721 | 19 | | |
| 70-89% | Ipsilateral | PS | 0.912 | 0.302 | 21 | 1.575 | 0.124 |
| 90-99% | Ipsilateral | PS | 0.759 | 0.313 | 19 | | |
| 70-89% | Contralateral | PS | 0.888 | 0.292 | 21 | 1.459 | 0.153 |
| 90-99% | Contralateral | PS | 0.746 | 0.322 | 19 | | |
| 70-89% | Ipsilateral | TTP | 24.002 | 2.213 | 21 | -0.199 | 0.843 |
| 90-99% | Ipsilateral | TTP | 24.209 | 4.009 | 19 | | |
| 70-89% | Contralateral | TTP | 23.905 | 2.102 | 21 | 0.135 | 0.893 |
| 90-99% | Contralateral | TTP | 23.762 | 4.147 | 19 | | |

CBF- Cerebral Blood Flow, CBV- Cerebral Blood Volume, MTT- Mean Transit Time, TTP- Time to Peak, PS- Permeability Surface Area-Product , SD- Standard Deviation.
